# Continuous Infusion and Sequential Nephron Blockade Versus Bolus Furosemide in Acute Heart Failure: A Systematic Review

**DOI:** 10.7759/cureus.111063

**Published:** 2026-06-17

**Authors:** Leena Awad Alkareem Ahmed Mohamed, Khalid Abozaid, Hala Fakhri MohammedAhmed Hassan, Yasir Ali, Nibras Elfatih Hussein Abdalla, Mohamed Samir Yasin Ibrahim, Weam Mohammed Ahmed Mohammed, Ahmed Mohammed Babikir Omer, Alaa Awad

**Affiliations:** 1 Internal Medicine, Al Artawiyah General Hospital, Riyadh, SAU; 2 General Internal Medicine, The Shrewsbury and Telford Hospital NHS Trust, Telford, GBR; 3 General Medicine, National Ribat University, Khartoum, SDN; 4 Acute Mecidine, Name Royal Stoke University Hospital, Staffordshire, GBR; 5 Dermatology, St. George’s University, St. George, GRD; 6 Pathophysiology, School of Medicine, St. George’s University, St. George, GRD; 7 Family Medicine, Primary Health Care, Khartoum, SDN; 8 Internal Medicine, Rustaq General Hospital, Ministry of Health, Alnahda, OMN; 9 Internal Medicine, Warrington and Halton Teaching Hospitals NHS Foundation Trust, Warrington, GBR

**Keywords:** acute heart failure, continuous infusion, diuretic resistance, furosemide, sequential nephron blockade, sglt2 inhibitors, systematic review

## Abstract

Acute heart failure (AHF) is a leading cause of hospitalization worldwide, with congestion as its central pathophysiologic feature. Loop diuretics, particularly furosemide, remain the cornerstone of decongestive therapy, yet the optimal administration strategy, continuous infusion versus bolus dosing, remains debated. Furthermore, sequential nephron blockade through the addition of distal tubule-acting diuretics or sodium-glucose cotransporter-2 (SGLT2) inhibitors has emerged as a strategy to overcome diuretic resistance. This systematic review had two primary objectives: first, to compare continuous infusion versus bolus dosing of furosemide; second, to evaluate the efficacy and safety of adjunctive sequential nephron blockade (SGLT2 inhibitors, thiazides, and acetazolamide) added to loop diuretics within the context of the ongoing debate over optimal decongestion in AHF.

A systematic literature search was conducted in PubMed, Scopus, Web of Science, the Cochrane Library, and ClinicalTrials.gov for studies published between 2021 and 2025. Eligible studies included randomized controlled trials (RCTs) and prospective observational studies evaluating either continuous furosemide infusion versus bolus furosemide or adjunctive sequential nephron blockade (added to loop diuretics) versus placebo, usual care, or, in one case, an active diuretic comparator. The Cochrane Risk of Bias 2 (RoB 2) tool was used for RCTs, and the Risk of Bias in Non-randomized Studies of Interventions (ROBINS-I) tool was used for the nonrandomized study. A narrative synthesis was performed because of substantial clinical and methodological heterogeneity.

Ten studies (nine RCTs and one prospective observational study) comprising 2,972 patients were included. Continuous furosemide infusion consistently improved surrogate measures of decongestion (urine output and weight loss) compared with bolus dosing. However, these benefits did not reliably translate into improved symptoms or shorter hospital stays, and one large study reported increased renal injury and adverse events, highlighting a potential efficacy-safety trade-off. Sequential nephron blockade with SGLT2 inhibitors enhanced diuresis with favorable renal and electrolyte safety profiles and a nonsignificant trend toward lower mortality (the studies were not powered for mortality). Thiazide-based strategies achieved potent diuresis but significantly increased the risks of acute kidney injury and electrolyte disturbances without a mortality benefit. Acetazolamide improved decongestion safely but did not reduce mortality or readmissions.

No single decongestive strategy is universally superior. However, direct comparisons across strategies are limited by substantial heterogeneity in study design, patient populations (e.g., renal function and congestion severity), and outcome definitions. Continuous furosemide infusion offers enhanced diuresis but inconsistent clinical benefits and potential renal harm. Based largely on surrogate outcomes from heterogeneous studies not designed to detect differences in mortality or readmissions, definitive clinical recommendations remain limited. SGLT2 inhibitors represent a promising but not yet proven adjunct in AHF, pending larger, adequately powered trials. Thiazide-based sequential blockade should be reserved for refractory cases with close monitoring. Treatment should be individualized based on baseline renal function, congestion severity, and electrolyte status.

## Introduction and background

Acute heart failure (AHF) is one of the most common causes of hospitalization worldwide and remains associated with high morbidity, mortality, and healthcare burden [1]. A central feature of AHF is systemic and pulmonary congestion due to fluid overload, which leads to symptoms such as shortness of breath, leg swelling, and reduced ability to perform daily activities [2]. Rapid and effective removal of excess fluid (decongestion) is therefore a primary treatment goal in the acute management of these patients.

Loop diuretics, particularly furosemide, are the mainstay of decongestive therapy in AHF [3]. These drugs work by blocking sodium reabsorption in the kidney, thereby increasing urine production. However, the optimal method of furosemide administration, either as intermittent bolus injections or as a continuous intravenous infusion, remains debated. Bolus injection is widely used because it is simple and works quickly, but it can cause fluctuations in drug levels in the blood, which may lead to poor response (diuretic resistance) and less effective sodium removal in some patients [4]. In contrast, continuous infusion keeps drug levels more stable, which may improve urine output and sodium removal and possibly reduce kidney stress. However, clinical studies have reported mixed results [5].

In patients who do not respond well to loop diuretics alone, a strategy called sequential nephron blockade has emerged as an important addition [6]. The concept is simple: instead of giving just one diuretic that targets a single part of the kidney tubule, adding a second diuretic that works on a different segment can overcome the kidney's natural tendency to compensate [7]. In this review, sequential nephron blockade includes three main classes of add-on medications: thiazides (e.g., hydrochlorothiazide and metolazone), acetazolamide (which acts on the proximal tubule), and sodium-glucose cotransporter-2 (SGLT2) inhibitors (e.g., dapagliflozin and empagliflozin). While this approach makes physiologic sense, the actual clinical benefits and safety of these different add-on drugs have been inconsistently reported.

Despite multiple randomized trials and observational studies comparing continuous infusion versus bolus furosemide and evaluating the addition of sequential nephron blockade, the evidence remains fragmented. Key sources of variation across studies include differences in patient populations (such as renal function, severity of congestion, and the presence of true diuretic resistance), differences in drug dosing (fixed versus adjusted based on response), inconsistent definitions of outcomes (for example, what constitutes worsening kidney function or successful decongestion), and varying lengths of follow-up. Major unresolved questions in the field include whether continuous infusion protects or harms the kidneys, whether adding a thiazide provides sufficient benefit to justify its safety risks, and whether newer drugs such as SGLT2 inhibitors have a clear role in the acute hospital setting. These ongoing controversies directly support the need for a systematic review.

Therefore, this systematic review had two separate but related objectives. First, to compare continuous furosemide infusion versus bolus furosemide dosing in patients with acute heart failure. Second, to evaluate the efficacy and safety of adding sequential nephron blockade (thiazides, acetazolamide, or SGLT2 inhibitors) to loop diuretics. These are distinct therapeutic strategies, and the review keeps them clearly separate. The review focused on key clinical outcomes, including the effectiveness of fluid removal, changes in kidney function, electrolyte disturbances, and in-hospital outcomes such as length of stay, with the goal of helping clinicians choose the most effective and safest decongestive strategy for their patients.

## Review

Methods

Study Design

This systematic review was conducted in accordance with the Preferred Reporting Items for Systematic Reviews and Meta-Analyses (PRISMA) guidelines [8] to ensure transparency, reproducibility, and methodological rigor. The review protocol was developed a priori, defining the research question, eligibility criteria, and planned methods of analysis before study selection and data extraction were undertaken. The protocol was not registered in PROSPERO or another public registry.

Eligibility Criteria (PICOS Framework)

The eligibility criteria were defined using the Population, Intervention, Comparison, Outcomes, and Study Design (PICOS) framework [9]. The population included patients with acute heart failure requiring intravenous diuretic therapy in hospital or emergency settings. This included adults with acute decompensated heart failure, as well as one pediatric study involving infants with left-to-right shunt and acute decompensated heart failure. The intervention consisted of continuous intravenous furosemide infusion or sequential nephron blockade strategies added to loop diuretic therapy. The comparator was intermittent or bolus intravenous furosemide administration. Outcomes of interest included measures of diuretic efficacy (urine output and weight reduction), clinical improvement (symptom relief and congestion resolution), renal outcomes (changes in serum creatinine and renal function parameters), electrolyte disturbances, and in-hospital outcomes such as length of stay and mortality. Only original research studies, including randomized controlled trials and observational cohort studies, were included, whereas reviews, editorials, case reports, and conference abstracts without full data were excluded. The study designs included under the "Study Design" component of PICOS were randomized controlled trials and prospective or retrospective comparative observational studies.

Information Sources

A comprehensive literature search was conducted in the following electronic databases: PubMed, Scopus, Web of Science, and the Cochrane Library. In addition, citation tracking and manual screening of reference lists of relevant articles were performed to identify additional eligible studies that may not have been captured through database searching. To further minimize publication bias and capture ongoing or unpublished evidence, ClinicalTrials.gov was also searched for registered clinical trials relevant to the topic.

Search Strategy

The search strategy combined controlled vocabulary terms and free-text keywords related to acute heart failure, furosemide, continuous infusion, bolus dosing, and sequential nephron blockade. Boolean operators ("AND" and "OR") were used to refine the search. The search was restricted to studies published in English between 2021 and 2025. The last search of these databases was performed on March 15, 2026. This time frame was deliberately selected to focus on contemporary evidence reflecting current clinical practice, updated guideline-directed therapy, and modern diuretic protocols. We acknowledge that older landmark studies remain clinically relevant. However, those earlier trials have been extensively reviewed and meta-analyzed in previous high-quality systematic reviews. Our review aims to provide an updated synthesis of the most recent evidence published after those influential trials, capturing new agents and evolving practice patterns. The full search strings for each database are provided in the Appendices.

Study Selection

All records retrieved from database searches were imported into EndNote X21 (Clarivate Plc, London, United Kingdom) for reference management and duplicate removal. After removing duplicate entries, titles and abstracts were independently screened for relevance based on the predefined eligibility criteria. Full-text articles of potentially eligible studies were then assessed in detail for final inclusion. Any disagreements between reviewers during the selection process were resolved through discussion and consensus. If consensus could not be reached, a third senior reviewer was consulted to adjudicate the disagreement.

Data Extraction

Data extraction was performed using a standardized and piloted data extraction form. Extracted variables included study characteristics (author, year, country, and study design), patient demographics, sample size, intervention and comparator details, diuretic strategy (continuous infusion, bolus dosing, or sequential nephron blockade), and relevant clinical outcomes such as urine output, weight change, symptom improvement, changes in renal function, electrolyte disturbances, and length of hospital stay.

Risk of Bias Assessment

The methodological quality of the included studies was assessed independently by two reviewers. Randomized controlled trials were evaluated using the Cochrane Risk of Bias 2 (RoB 2) tool [10], whereas nonrandomized observational studies were assessed using the Risk of Bias in Non-randomized Studies of Interventions (ROBINS-I) tool [11]. These instruments were chosen to ensure a robust and design-specific assessment of bias across different study types, focusing on domains such as confounding, selection bias, intervention classification, missing data, and outcome measurement.

Data Synthesis and Statistical Analysis

A narrative synthesis approach was adopted to summarize the findings across the included studies, following the Synthesis Without Meta-analysis (SWiM) reporting guidelines. Studies were grouped by intervention type into four clinically coherent categories: continuous furosemide infusion versus bolus dosing, SGLT2 inhibitor add-on therapy, thiazide-based sequential blockade, and acetazolamide. Within each category, results were summarized descriptively, highlighting patterns of benefit, inconsistencies across studies, and differences in clinical outcomes. Due to substantial heterogeneity in study designs, patient populations, intervention protocols, and outcome definitions, a quantitative meta-analysis was not performed. Pooling such heterogeneous data could produce misleading effect estimates and reduce the validity of the conclusions. No GRADE certainty assessment was performed because of the narrative design and lack of pooled effect estimates.

Justification for Lack of Meta-analysis

A meta-analysis was deemed inappropriate because of significant clinical and methodological heterogeneity among the included studies. Examples of this heterogeneity include variability in furosemide dosing protocols, such as fixed doses versus protocolized titration, and differences in adjunctive agents, including dapagliflozin, empagliflozin, hydrochlorothiazide, metolazone, and acetazolamide. Outcome definitions also varied considerably, with decongestion measured as urine output, weight loss, or clinical congestion scores, and renal impairment defined as serum creatinine increases ranging from 0.3 mg/dL to 50% increases or as declines in eGFR. Follow-up durations ranged from 48 hours to 180 days, and baseline patient severity differed across studies in terms of ejection fraction, renal function, and diuretic resistance status. Limited subgroup analyses or quantitative pooling were considered but not pursued because, even within potential subgroups, such as studies evaluating continuous infusion only, heterogeneity in dosing protocols and outcome definitions remained substantial. As a result, a structured narrative synthesis by intervention category was considered the most appropriate method for providing a meaningful and clinically interpretable summary without introducing statistical bias from inappropriate data pooling.

Results

Studies Selection Process

**Figure 1:**
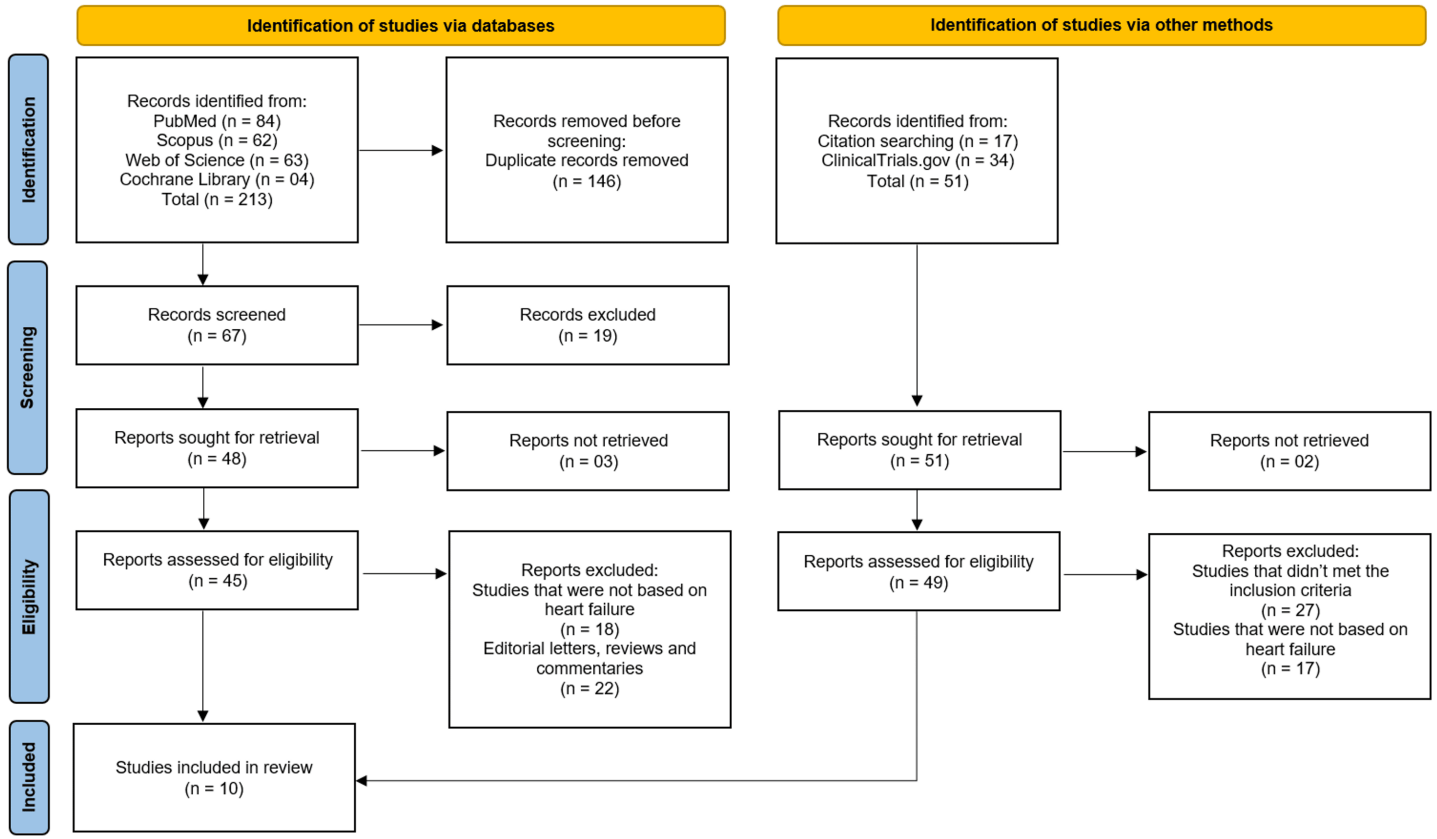
PRISMA Flowchart PRISMA: Preferred Reporting Items for Systematic Reviews and Meta-Analyses.

The study selection process followed the PRISMA guidelines and is summarized in Figure 1. A total of 213 records were identified through database searches, including PubMed (n = 84), Scopus (n = 62), Web of Science (n = 63), and the Cochrane Library (n = 4). An additional 51 records were identified through other methods, comprising citation searching (n = 17) and ClinicalTrials.gov (n = 34), bringing the total number of identified records to 264. After duplicate records were removed (n = 146), 118 records remained for screening. Of these, 67 records were from databases and 51 were from other methods. Following screening, 19 records were excluded, leaving 99 reports sought for retrieval (51 from databases and 48 from other methods). Of these, two reports from databases and three reports from other methods could not be retrieved, resulting in 49 reports from databases and 45 reports from other methods being assessed for eligibility. During eligibility assessment, 18 reports from databases were excluded because they were not related to heart failure, and 22 editorial letters, reviews, and commentaries were also excluded. Among reports identified through other methods, 27 were excluded for not meeting the inclusion criteria and 17 were excluded because they were not related to heart failure. Ultimately, 10 studies [12-21] met the eligibility criteria and were included in this systematic review. 

Characteristics of Included Studies

A total of 10 studies met the inclusion criteria for this systematic review, comprising a mix of RCTs and one prospective observational study. Their key characteristics are summarized in Table 1. The studies were conducted across diverse geographical settings, including Italy, the USA, Pakistan, the UK, Egypt, Spain, Germany, Belgium, Brazil, and China [12-21]. Sample sizes ranged from 51 to 1,276 patients, with the majority focusing on hospitalized adults with acute decompensated heart failure (ADHF); one trial specifically included infants with left-to-right shunt in a pediatric intensive care unit [16]. The interventions varied widely: three studies compared continuous intravenous (IV) furosemide infusion versus bolus administration [12,14,21], whereas others evaluated sequential nephron blockade using SGLT2 inhibitors (dapagliflozin or empagliflozin) [13,15,18], thiazides (hydrochlorothiazide) [17,20], or acetazolamide [19]. One study also evaluated hypertonic saline added to loop diuretics [14]. Follow-up durations ranged from 48 hours during hospitalization to 180 days postdischarge. Sequential nephron blockade agents included dapagliflozin, empagliflozin, metolazone, hydrochlorothiazide, and acetazolamide [13,15,17-20]. 

**Table 1:** Characteristics of Included Studies AHF, acute heart failure; ADHF, acute decompensated heart failure; HFrEF, heart failure with reduced ejection fraction; AF, atrial fibrillation; CHD, congenital heart disease; L→R, left-to-right; PICU, pediatric intensive care unit; RCT, randomized controlled trial; DB, double-blind; PC, placebo-controlled; IV, intravenous; CI, continuous infusion; CIVI, continuous intravenous infusion; HSS, hypertonic saline solution; HCTZ, hydrochlorothiazide; OD, once daily; q8h, every 8 hours; mg/kg/h, milligrams per kilogram per hour; eGFR, estimated glomerular filtration rate; NYHA, New York Heart Association; FU, follow-up.

Author (Year)	Country	Study Design	Sample Size	Population/Setting	Intervention Group	Comparator Group	Furosemide Strategy	Sequential Nephron Blockade Agent	Follow-up Duration
Palazzuoli et al., [12] (2025)	Italy	Multicenter prospective observational trial	402 AHF patients	Hospitalized AHF patients in 6 HF centers	Continuous IV furosemide (n=197)	Bolus IV furosemide (n = 205)	Continuous vs twice-daily bolus IV infusion (72-120 h)	None	180 days
Cox et al., [13] (2024)	USA	Multicenter RCT, open-label	238	Hospitalized AHF adults on IV loop diuretics	Dapagliflozin + IV loop diuretics	Usual care + IV loop diuretics	IV furosemide protocol (bolus/infusion, titrated)	Dapagliflozin	5 days/discharge + 30 days
Khan et al., [14] (2024)	Pakistan	Prospective RCT (3-arm)	1276	ADHF + HFrEF + AF (inpatients)	CI + HSS	Bolus IV furosemide	Bolus vs CI vs HSS combo	Hypertonic saline (1.95%)	48 hours (in-hospital)
Yeoh et al., [15] (2023)	UK	Multicenter RCT	61 (60)	AHF, diuretic-resistant inpatients	Dapagliflozin 10 mg OD	Metolazone 5-10 mg OD	IV loop ≥160 mg/day pre-rand	Metolazone	5 days + 90 days
Zarzor et al., [16] (2023)	Egypt	RCT (open-label)	54 (27/27)	Infants with ADHF (CHD, L→R shunt), PICU	IV furosemide infusion	IV bolus q8h	CIVI 0.125-0.25 mg/kg/h vs bolus 1-2 mg/kg q8h	None	49 hours + 30 days FU
Trullàs et al., [17] (2023)	Spain	RCT, DB, PC	230	ADHF, ≤24 h admit	IV furosemide + HCTZ	IV furosemide + placebo	Protocolized IV loop (low-dose)	HCTZ	5 days + 90 days
Schulze et al., [18] (2022)	Germany	DB-RCT	60 (30/30)	ADHF, hospitalized	Empagliflozin 25 mg	Placebo	Usual loop diuretics	None	5 days (+30 days FU)
Mullens et al., [19] (2022)	Belgium (27 centers)	DB-RCT	519 (259 vs 260)	Hospitalized ADHF with congestion + elevated NT-proBNP	IV acetazolamide + standard loop diuretics	Placebo + standard loop diuretics	IV loop diuretic bolus = 2× oral maintenance; split dosing	Acetazolamide	3 months
Piardi et al., [20] (2021)	Brazil	RCT, double-blind	51 (26/25)	ADHF, EF ≤45%, ED patients	HCTZ 50 mg + IV furosemide	Placebo + IV furosemide	IV, clinician-guided	HCTZ	3 days/discharge
Zheng et al., [21] (2021)	China	RCT (prospective)	81	ADHF + CKD (NYHA III-IV, eGFR 15-44.9), hospital	IV continuous infusion furosemide	IV bolus furosemide	Fixed dose IV (160-200 mg/day) CI vs BI	None	72 hours

Efficacy Outcomes: Urine Output, Diuresis, and Weight Loss

Continuous furosemide infusion consistently improved diuresis compared with bolus dosing. Palazzuoli et al. reported higher urine output (2181 vs 2019 mL) and greater weight loss (4.3 vs 3.2 kg) with continuous infusion, although this was associated with increased renal injury [12]. Similarly, Zheng et al. found significantly higher urine output (5146 vs 3756 mL, p = 0.01) and weight loss (4.72 vs 3.53 kg) with continuous infusion, along with better decongestion and a shorter hospital stay [21]. In the pediatric population, Zarzor et al. observed superior urine output and weight loss with continuous infusion versus bolus dosing, with comparable safety [16]. Khan et al. reported numerically greater weight loss with continuous infusion (5.7 kg) compared with bolus dosing (4.1 kg) and hypertonic saline (4.6 kg), but the differences were not statistically significant; however, bolus dosing significantly reduced length of stay [14].

Regarding sequential nephron blockade, Cox et al. demonstrated that adding dapagliflozin to loop diuretics increased urine output per 40 mg of furosemide (634 vs 403 mL, p = 0.005) and sodium excretion (p = 0.025), with similar weight loss between groups [13]. Schulze et al. found that empagliflozin significantly increased total urine output (10.8 vs 8.65 L, p = 0.003), with a nonsignificant trend toward greater weight loss [18]. Trullàs et al. reported that adding hydrochlorothiazide to furosemide increased 24-hour urine output (1775 vs 1400 mL) and weight loss (−2.5 vs −1.5 kg) [17]. Yeoh et al. noted similar urine output and net fluid balance between dapagliflozin and metolazone, although metolazone produced slightly greater weight loss and stronger natriuresis at the cost of worse renal and electrolyte profiles [15]. Mullens et al. found that acetazolamide increased 48-hour urine output (4.6 vs 4.1 L) and decongestion rates (42% vs 30%) without reporting significant weight loss data [19]. Piardi et al. reported an improved diuretic response per furosemide dose with hydrochlorothiazide (−0.74 vs −0.33 kg/40 mg, p = 0.032) and greater weight loss that did not reach statistical significance [20].

Symptom Improvement and Decongestion

Most studies reported no major differences in patient-reported symptom improvement between intervention and comparator groups. Palazzuoli et al. found similar congestion scores between continuous and bolus furosemide administration [12], and Cox et al. reported comparable symptom relief with slight congestion reduction in both arms [13]. Zarzor et al. noted faster clinical improvement with continuous infusion in infants [16]. Trullàs et al. observed no difference in visual analog scale or Likert scale scores [17]. Schulze et al. reported improved New York Heart Association class with empagliflozin despite no change in quality of life [18]. Mullens et al. found significantly better decongestion with acetazolamide (42% vs 30%) [19], and Zheng et al. reported greater improvement in Borg scores and better decongestion with continuous furosemide infusion [21]. Yeoh et al. noted similar congestion improvement between dapagliflozin and metolazone based on lung ultrasound, edema, and symptom scores [15].

Length of Hospital Stay

Continuous furosemide infusion was associated with differences in hospital stay across studies. Palazzuoli et al. reported a longer hospital stay with continuous infusion (12.7 vs 11.5 days) [12], whereas Zheng et al. found a significantly shorter hospital stay with continuous infusion (10.4 vs 15.7 days) [21]. Khan et al. reported that bolus furosemide reduced length of stay (3.7 days vs 6.6 days for continuous infusion and 7.9 days for hypertonic saline) [14]. In trials evaluating sequential nephron blockade, Cox et al. noted a nonsignificant trend toward a shorter hospital stay with dapagliflozin (5.0 vs 6.2 days) [13], and Mullens et al. found a shorter hospital stay with acetazolamide (8.8 vs 9.9 days) [19]. Trullàs et al. and Yeoh et al. reported no difference in hospital stay between intervention groups [15,17]. Piardi et al. also found no difference [20].

Renal Function and Electrolyte Disturbances

Renal safety varied considerably across interventions. Continuous furosemide infusion was associated with higher rates of worsening renal function (WRF) in the study by Palazzuoli et al. (35% vs 18%) and more frequent hypokalemia [12]. However, Zheng et al. found similar rates of acute kidney injury (AKI) between continuous and bolus groups (~17% vs 23%) and no significant electrolyte differences [21]. Zarzor et al. reported mild, nonsignificant increases in creatinine with continuous infusion [16]. Among studies evaluating sequential nephron blockade, Trullàs et al. reported significantly higher rates of AKI with hydrochlorothiazide (46.5% vs 17.2%) and more hypokalemia, although there was no difference in hyponatremia [17]. Yeoh et al. found that metolazone increased creatinine and reduced eGFR more than dapagliflozin, with more dysnatremia and electrolyte shifts [15]. In contrast, Cox et al. reported minimal changes in eGFR (−2.0 vs −3.2) and no major electrolyte imbalance with dapagliflozin [13]. Schulze et al. observed fewer AKI events and stable eGFR with empagliflozin [18]. Mullens et al. reported no AKI signal with acetazolamide [19]. Piardi et al. found a borderline increase in creatinine with hydrochlorothiazide but no significant difference in WRF [20]. Khan et al. reported no significant differences in creatinine or electrolyte levels between groups [14].

Mortality and Readmission

Palazzuoli et al. reported higher mortality or heart failure readmission rates with continuous furosemide infusion (45% vs 29%) and more hemodialysis events [12]. Cox et al. noted fewer deaths with dapagliflozin (1 vs 5) and similar readmission rates [13]. Yeoh et al. reported in-hospital mortality rates of 7% with dapagliflozin versus 13% with metolazone and 90-day mortality rates of 17% versus 23%, with similar readmission rates [15]. Trullàs et al. found no difference in 30- or 90-day mortality or readmission rates [17]. Schulze et al. reported lower mortality with empagliflozin (3.3% vs 6.9%) [18]. Mullens et al. found no significant difference in the composite outcome of death or readmission (29.7% vs 27.8%) [19]. Piardi et al. reported no difference in mortality and did not report readmission data [20]. Zheng et al. reported no deaths in either group [21]. Khan et al. did not report mortality or readmission outcomes [14]. Zarzor et al. found no significant differences in mortality [16].

Summary of Main Conclusions Across Studies

As shown in Table 2, the main conclusions of the included studies were heterogeneous. Continuous furosemide infusion generally improved diuresis and weight loss but was associated with increased renal injury and adverse events in some trials [12,21]. Sequential nephron blockade with SGLT2 inhibitors (dapagliflozin and empagliflozin) provided better diuresis with favorable or neutral renal safety profiles [13,18]. Thiazide-based strategies (hydrochlorothiazide and metolazone) enhanced diuretic response but consistently increased the risks of AKI and electrolyte disturbances without a clear mortality benefit [15,17,20]. Acetazolamide improved decongestion but did not reduce mortality or readmission [19]. Overall, no single strategy demonstrated superiority across all efficacy and safety domains, highlighting the need for patient-tailored approaches in the management of acute heart failure.

**Table 2:** Clinical Outcomes and Efficacy Findings ↑, increase or higher; ↓, decrease or lower; vs, versus; CI, continuous infusion; cIV, continuous intravenous infusion; bI, bolus injection; HD, hemodialysis; WRF, worsening renal function; eGFR, estimated glomerular filtration rate; AKI, acute kidney injury; UO, urine output; Na, sodium; NS, not significant; NR, not reported; LOS, length of stay; HSS, hypertonic saline solution; LUS, lung ultrasound; hypoK, hypokalemia; hypoNa, hyponatremia; WL, weight loss; VAS, visual analog scale; QoL, quality of life; NYHA, New York Heart Association; NT-proBNP, N-terminal pro-b-type natriuretic peptide; CI, confidence interval; Cr, creatinine; d, days; h, hours; kg, kilograms; L, liters; mL, milliliters; mg, milligrams; mmol, millimoles; kg/40 mg, weight loss per 40 milligrams of furosemide.

Author (Year)	Urine Output/Diuresis	Weight Loss	Symptom Improvement	Length of Hospital Stay	Renal Function Changes	Electrolyte Disturbances	Mortality/Readmission	Main Conclusion
Palazzuoli et al., [12] (2025)	↑ CiV (2181 vs 2019 mL); HD ↑	↑ CiV (4.3 vs 3.2 kg)	Similar; no diff in congestion score	↑ CiV (12.7 vs 11.5 d)	↑ WRF CiV (35% vs 18%); ↓ eGFR	↑ hypokalemia CiV; more imbalance	↑ CiV (45% vs 29%); ↑ HD events	CiV/HD ↑ diuresis but ↑ renal injury & events vs bolus
Cox et al., [13] (2024)	↑ UO: 634 vs 403 mL/40 mg; ↑ Na excretion (p = 0.005, 0.025)	Similar: –4.0 vs –4.2 kg	Similar; slight ↓ congestion; ↓ NT-proBNP both	5.0 vs 6.2 d (NS); ↑ early discharge	eGFR: –2.0 vs –3.2 (NS)	K+ <3.0: 1 vs 1; no major imbalance	Death 1 vs 5; readmission similar	Better diuresis & lower loop dose, no safety harm
Khan et al., [14] (2024)	NR	cIV 5.7 kg; bI 4.1 kg; HSS 4.6 kg (NS)	NR	cIV 3.7 d; bI 6.6 d; HSS 7.9 d	No sig diff; Cr change NS; 48h Cr slight diff	Na/K NS between groups	NR	bI reduced LOS vs cIV/HSS; no diff in weight, renal, electrolytes
Yeoh et al., [15] (2023)	Similar UO & net balance; ↑ urinary Na with metolazone	Slightly ↑ with metolazone (−3.6 vs −3.0 kg; NS)	Similar congestion improvement (LUS, edema, scores)	20 vs 19 days (NS)	Metolazone ↑Cr/↓eGFR; dapagliflozin more stable	Metolazone ↓Na, more dysnatremia; more shifts overall	7% vs 13% in-hospital; 17% vs 23% at 90d; similar readmission	Both strategies similar efficacy; metolazone stronger diuresis but worse renal/electrolyte profile
Zarzor et al., [16] (2023)	CIVI ↑ UOP vs bolus	CIVI > loss	Faster CIVI	NS	Mild ↑Cr CIVI (normal)	NS	NS	CIVI superior efficacy, similar safety
Trullàs et al., [17] (2023)	↑ 24h UO (1775 vs 1400 mL)	↑ WL (−2.5 vs −1.5 kg)	No diff (VAS/Likert NS)	7 vs 7 days (NS)	↑ AKI (46.5% vs 17.2%)	↑ HypoK; no major hypoNa diff	No diff (30/90d death & readm)	↑ diuresis & WL but ↑ renal + hypoK, no outcome benefit
Schulze et al., [18] (2022)	↑ UO (10.8 vs 8.65 L, p = 0.003)	−4.2 vs −3.0 kg (NS)	↑ NYHA; QoL NS	NS diff reported	↓ AKI events; eGFR stable	No major imbalance	3.3% vs 6.9% mortality	Empagliflozin ↑ diuresis, safe renal profile
Mullens et al., [19] (2022)	4.6 L vs 4.1 L ↑	NR	Decongestion ↑ (42% vs 30%)	8.8 vs 9.9 days ↓	Similar; no AKI signal	No severe changes	29.7% vs 27.8% (NS)	↑ decongestion; no outcome benefit
Piardi et al., [20] (2021)	Improved diuretic response per furosemide dose (−0.74 vs −0.33 kg/40 mg; p = 0.032)	Greater but NS (−1.78 vs −1.05 kg/day; p = 0.062)	No difference (dyspnea/congestion/thirst)	No difference	↑ creatinine borderline; WRF NS (p = 0.38)	No differences	No mortality difference; no readmission data	HCTZ improved diuretic response but no clinical outcome benefit
Zheng et al., [21] (2021)	CI ↑ (5146 vs 3756 mL, p = 0.01)	CI ↑ (4.72 vs 3.53 kg)	CI better (↓Borg, ↑decongestion)	CI shorter (10.4 vs 15.7 d)	Similar (AKI ~17% vs 23%)	No sig. diff	0 deaths; no data	CI superior diuresis, decongestion & LOS; similar safety

Risk of Bias Assessment

The risk of bias for the nine randomized controlled trials was assessed using the Cochrane RoB 2 tool, whereas the single nonrandomized prospective observational study [12] was evaluated using the ROBINS-I tool, as summarized in Tables 3 and 4. Among the RCTs, seven studies [13-15,17-20] demonstrated a low risk of bias across all five domains. Two open-label RCTs had some concerns because of the lack of blinding. The study by Zarzor et al. had some concerns in the domain of deviations from intended interventions, whereas the study by Zheng et al. had some concerns in both deviations from intended interventions and outcome measurement, primarily because subjective outcomes such as symptom scores may have been influenced by the absence of blinding [16,21]. The nonrandomized study by Palazzuoli et al. was assessed using the ROBINS-I tool and had an overall moderate risk of bias, with moderate concerns related to confounding and outcome measurement but a low risk of bias in the classification of interventions and missing data domains [12]. No study was excluded from the review based on risk of bias, although findings from the moderate-risk observational study and the two RCTs with some concerns should be interpreted with appropriate caution (Tables 3, 4). 

**Table 3:** Risk of Bias Assessment for Non-Randomized Study Using ROBINS-I Tool

Author (Year)	Bias due to Confounding	Bias in Selection of Participants	Bias in Classification of Interventions	Bias due to Missing Data	Bias in Measurement of Outcomes	Bias in Selection of Reported Result	Overall Risk of Bias
Palazzuoli et al., [12] (2025)	Moderate	Low	Low	Low	Moderate	Moderate	Moderate

**Table 4:** Risk of Bias Assessment for RCTs Using Cochrane RoB 2 Tool

Author (Year)	Randomization Process	Deviations from Intended Interventions	Missing Outcome Data	Measurement of the Outcome	Selection of Reported Result	Overall Risk of Bias
Cox et al., [13] (2024)	Low	Low	Low	Low	Low	Low
Khan et al., [14] (2024)	Low	Low	Low	Low	Low	Low
Yeoh et al., [15] (2023)	Low	Low	Low	Low	Low	Low
Zarzor et al., [16] (2023)	Low	Some concerns	Low	Low	Low	Some concerns
Trullàs et al., [17] (2023)	Low	Low	Low	Low	Low	Low
Schulze et al., [18] (2022)	Low	Low	Low	Low	Low	Low
Mullens et al., [19] (2022)	Low	Low	Low	Low	Low	Low
Piardi et al., [20] (2021)	Low	Low	Low	Low	Low	Low
Zheng et al., [21] (2021)	Low	Some concerns	Low	Some concerns	Low	Some concerns

Discussion

The findings of this systematic review provide a comprehensive synthesis of current evidence comparing continuous furosemide infusion versus bolus administration, as well as sequential nephron blockade strategies, in patients with acute heart failure. Across 10 heterogeneous studies, several important patterns emerge that have direct implications for clinical practice and future research. First, continuous furosemide infusion consistently achieved superior diuresis and weight loss compared with bolus dosing [12,16,21]. This observation aligns with the pharmacokinetic rationale that continuous infusion avoids the peak-trough fluctuations associated with intermittent boluses, thereby maintaining more consistent loop diuretic concentrations at the nephron's thick ascending limb [22]. However, the enhanced diuretic efficacy of continuous infusion did not translate uniformly into improved clinical outcomes. Notably, Palazzuoli et al. reported paradoxically longer hospital stays and higher rates of worsening renal function and mortality or readmission with continuous infusion [12], whereas Zheng et al. found shorter hospital stays and similar renal safety [21]. These discrepant findings likely reflect differences in patient populations. Zheng et al. specifically enrolled patients with moderate chronic kidney disease (eGFR, 15-44.9 mL/min), who may have different diuretic handling and renal reserve compared with the broader heart failure population in the study by Palazzuoli et al. [12]. This heterogeneity suggests that the optimal furosemide administration strategy may depend on baseline renal function.

Sequential nephron blockade, the addition of a distal tubule-acting diuretic or an SGLT2 inhibitor to a loop diuretic, emerged as a powerful strategy to overcome diuretic resistance. The SGLT2 inhibitors dapagliflozin and empagliflozin consistently increased urine output and sodium excretion without major renal safety signals [13,18]. These findings extend the known benefits of SGLT2 inhibitors from chronic heart failure to the acute setting, a transition that has been cautiously embraced following large trials such as DAPA-HF [23], which established their long-term efficacy. The observation by Cox et al. that dapagliflozin allowed lower loop diuretic doses while maintaining diuresis is clinically valuable because high-dose loop diuretics are independently associated with neurohormonal activation and adverse outcomes [13]. Schulze et al. further demonstrated that early empagliflozin initiation reduced AKI events compared with placebo, challenging the traditional concern that SGLT2 inhibitors might precipitate volume depletion or kidney injury in acutely decompensated patients [18]. These data collectively support a paradigm shift: rather than escalating loop diuretic doses in resistant patients, clinicians might consider early addition of an SGLT2 inhibitor, provided that hemodynamic stability and adequate perfusion pressure are ensured.

In contrast, thiazide-based sequential nephron blockade using hydrochlorothiazide or metolazone produced more concerning safety profiles despite superior diuresis. Trullàs et al. reported that adding hydrochlorothiazide to furosemide almost tripled the risk of AKI (46.5% vs 17.2%) and significantly increased hypokalemia [17]. Similarly, Yeoh et al. found that metolazone caused greater creatinine elevation and more frequent dysnatremia and electrolyte shifts compared with dapagliflozin [15]. Piardi et al. also observed a borderline increase in creatinine with hydrochlorothiazide without a clinical outcome benefit [20]. These findings are consistent with older literature showing that thiazide-loop combination therapy, while effective for diuretic resistance, carries substantial risks of electrolyte disturbances and renal impairment [24]. The absence of a mortality or readmission benefit in any thiazide trial included in this review [15,17,20] suggests that the risks of thiazide addition may outweigh the benefits except in carefully selected, closely monitored patients with refractory congestion. Acetazolamide, a proximal tubule carbonic anhydrase inhibitor, offered an intermediate profile. Mullens et al. demonstrated improved decongestion and a trend toward a shorter hospital stay without a significant AKI signal, but no reduction in death or readmission [19].

One of the most striking findings of this review is the dissociation between diuretic efficacy and patient-important outcomes. Multiple interventions, including continuous infusion, thiazides, and even acetazolamide, increased urine output and weight loss but failed to reduce mortality or readmissions or to consistently shorten hospital stays [12,14,17,19,20]. This phenomenon has been observed in prior heart failure trials, where aggressive decongestion sometimes led to renal injury that offset any potential benefit [25]. The study by Palazzuoli et al. exemplifies this trade-off: continuous infusion produced greater diuresis but also higher rates of worsening renal function and adverse events, resulting in net harm [12]. These data reinforce the concept that diuresis is a surrogate endpoint, not a substitute for hard clinical outcomes. From a clinical perspective, the goal should be euvolemia achieved safely, not maximal urine output.

Mortality and readmission data across the included studies were inconsistent. Cox et al. reported numerically fewer deaths with dapagliflozin (1 vs 5), and Schulze et al. reported lower mortality with empagliflozin (3.3% vs 6.9%). However, neither trial was powered to detect differences in mortality, so these findings should be considered hypothesis-generating only and require confirmation in larger, adequately powered trials [13,18]. The largest mortality dataset came from the study by Palazzuoli et al., which showed harm with continuous infusion [12]. Trullàs et al. and Mullens et al. found no difference in mortality with hydrochlorothiazide or acetazolamide, respectively [17,19]. These observations are consistent with a recent meta-analysis by Ul Amin et al., which concluded that loop diuretic administration strategy does not independently influence mortality in acute heart failure but that SGLT2 inhibitors may offer a mortality benefit that requires confirmation in larger dedicated trials [26]. Importantly, no study in this review demonstrated a mortality advantage for any intervention, highlighting that decongestion strategies should be evaluated primarily on safety, symptom relief, and cost-effectiveness unless a survival benefit is proven.

Renal safety emerged as a critical differentiator between strategies. Continuous infusion had variable effects: Palazzuoli et al. reported harm, whereas Zheng et al. and Zarzor et al. found comparable safety to bolus dosing [12,16,21]. This discrepancy may relate to patient selection and differences in the definition of worsening renal function (e.g., serum creatinine, eGFR, or urinary biomarkers). SGLT2 inhibitors, in contrast, consistently demonstrated renal protection or neutrality [13,18], aligning with their known renoprotective effects mediated by tubuloglomerular feedback and reduced intraglomerular pressure. Thiazides consistently impaired renal function [15,17,20], which may be explained by their prolonged duration of action and greater electrolyte disturbances leading to prerenal physiology. Based on the available data, a tentative interpretive summary might suggest the following safety pattern: SGLT2 inhibitors appeared most favorable, followed by acetazolamide, whereas continuous infusion showed mixed results and thiazides consistently raised safety concerns. However, given the heterogeneity across studies, this should not be interpreted as a definitive clinical ranking.

Electrolyte disturbances, particularly hypokalemia, were common with thiazide-based strategies [15,17] and, to a lesser extent, with continuous infusion [12]. Hypokalemia is not a benign laboratory finding; it increases the risk of ventricular arrhythmias and sudden death in heart failure patients, especially those receiving digoxin or those with reduced ejection fraction [27]. The absence of routine potassium monitoring protocols or standardized supplementation in most included studies is a notable gap. Future trials should incorporate predefined electrolyte management algorithms.

From a health systems perspective, continuous infusion requires intravenous access, infusion pumps, and dedicated nursing time, whereas bolus dosing is simpler and less costly. Despite the potential diuretic advantage of continuous infusion, the lack of consistent clinical benefit and the signal for harm in one large study argue against its routine use [12]. However, the positive results reported by Zheng et al. in the CKD subgroup [21] suggest that continuous infusion may be beneficial in specific phenotypes, a hypothesis that warrants prospective validation. Similarly, SGLT2 inhibitors are administered orally, are convenient, and are now available as generic medications in many countries, making them attractive add-on therapies.

Several mechanisms may explain the observed heterogeneity. First, baseline renal function, diuretic naivety, and the degree of congestion varied widely across studies. Second, the definition of "bolus" was not standardized; some studies used fixed low doses, whereas others titrated treatment according to response. Third, concomitant medications such as beta-blockers, ACE inhibitors, and mineralocorticoid receptor antagonists were inconsistently reported. Fourth, the timing of outcome assessment differed, with some studies capturing in-hospital events and others extending follow-up to 180 days. Finally, the open-label design of many studies [13-16,21] introduces performance and detection bias for subjective endpoints, although objective endpoints such as urine output and mortality are less susceptible to such bias.

The findings of this review must be interpreted in the context of the evolving understanding of heart failure pathophysiology. The classic model of "diuretic resistance" as simply a pharmacokinetic problem is being replaced by a more nuanced view that includes neurohormonal activation, renal venous congestion, and tubular tolerance [28]. SGLT2 inhibitors may address multiple pathways simultaneously by enhancing natriuresis, reducing intraglomerular pressure, and modulating inflammation, which could explain their favorable renal safety profile despite potent diuretic effects. In contrast, thiazides may produce "overdiuresis" without these protective mechanisms, leading to renal injury.

Limitations

Several limitations of this systematic review must be acknowledged. First, the included studies exhibited substantial clinical and methodological heterogeneity, precluding a meta-analysis. Differences in patient populations (adults vs infants, preserved vs reduced ejection fraction, presence or absence of CKD), interventions (dose, duration, route), comparators (placebo vs usual care vs active diuretic), and outcomes prevented pooling of results. Second, the majority of studies were open-label, introducing potential bias in the assessment of subjective endpoints, although objective outcomes were less affected. Third, the single nonrandomized study [12] had a moderate risk of bias because of confounding and should be interpreted cautiously. Fourth, sample sizes were modest for most efficacy outcomes, particularly mortality, leading to wide confidence intervals and a risk of type II error. Fifth, publication bias cannot be excluded; studies with negative or null results may be underrepresented. Sixth, the review did not include unpublished data or gray literature beyond ClinicalTrials.gov. Seventh, most studies had short follow-up durations (48 hours to 90 days), limiting assessment of long-term safety and efficacy. Eighth, patient-reported outcomes and quality-of-life measures were inconsistently reported despite their importance in heart failure management. Finally, the search was limited to English-language publications, which may introduce language bias.

## Conclusions

Continuous furosemide infusion produces greater diuresis than bolus administration but does not consistently improve clinical outcomes and may increase renal injury in some populations. Sequential nephron blockade with SGLT2 inhibitors (dapagliflozin and empagliflozin) offers a promising strategy: enhanced diuresis with favorable renal and electrolyte safety profiles, and a hypothesis-generating signal toward lower mortality that requires confirmation in adequately powered trials. In contrast, thiazide-based sequential blockade (hydrochlorothiazide and metolazone) achieves potent diuresis at the cost of significantly higher rates of AKI and electrolyte disturbances, without demonstrable outcome benefit. Acetazolamide occupies an intermediate position, improving decongestion safely but without reducing mortality. No single strategy is universally superior; treatment should be individualized based on baseline renal function, congestion severity, diuretic resistance, and electrolyte status. Future large, double-blind, pragmatic trials with protocolized diuretic titration, standardized electrolyte management, and long-term follow-up are needed to define optimal decongestion strategies. For now, available evidence suggests that early addition of SGLT2 inhibitors may be considered in eligible patients with acute heart failure, whereas thiazide combinations should generally be avoided except in refractory cases with close monitoring, and continuous infusion should be reserved for selected patients in whom bolus dosing has failed and renal function is stable. The ultimate goal remains safe, effective decongestion that improves symptoms without compromising renal function or electrolyte balance, a balance that this review suggests may be more achievable with SGLT2 inhibitors than with traditional diuretic intensification strategies, although direct comparative evidence remains limited.

## Appendices

**Table 5:** Full Search Strings for Databases

Database	Search String
PubMed	("acute heart failure"[MeSH Terms] OR "acute decompensated heart failure"[Title/Abstract] OR "ADHF"[Title/Abstract] OR "acute heart failure"[Title/Abstract]) AND (furosemide[MeSH Terms] OR furosemide[Title/Abstract] OR "loop diuretic"[Title/Abstract]) AND ("continuous infusion"[Title/Abstract] OR "continuous intravenous"[Title/Abstract] OR "bolus"[Title/Abstract] OR "intermittent"[Title/Abstract] OR "sequential nephron blockade"[Title/Abstract] OR "SGLT2 inhibitor"[Title/Abstract] OR "dapagliflozin"[Title/Abstract] OR "empagliflozin"[Title/Abstract] OR "thiazide"[Title/Abstract] OR "hydrochlorothiazide"[Title/Abstract] OR "metolazone"[Title/Abstract] OR "acetazolamide"[Title/Abstract]) AND (randomized controlled trial[Publication Type] OR controlled clinical trial[Publication Type] OR observational study[Publication Type]) AND (2021/01/01:2025/12/31[Date - Publication]) AND english[Language]
Scopus	( TITLE-ABS-KEY("acute heart failure") OR TITLE-ABS-KEY("acute decompensated heart failure") OR TITLE-ABS-KEY("ADHF") ) AND ( TITLE-ABS-KEY("furosemide") OR TITLE-ABS-KEY("loop diuretic") ) AND ( TITLE-ABS-KEY("continuous infusion") OR TITLE-ABS-KEY("continuous intravenous") OR TITLE-ABS-KEY("bolus") OR TITLE-ABS-KEY("intermittent") OR TITLE-ABS-KEY("sequential nephron blockade") OR TITLE-ABS-KEY("SGLT2 inhibitor") OR TITLE-ABS-KEY("dapagliflozin") OR TITLE-ABS-KEY("empagliflozin") OR TITLE-ABS-KEY("thiazide") OR TITLE-ABS-KEY("hydrochlorothiazide") OR TITLE-ABS-KEY("metolazone") OR TITLE-ABS-KEY("acetazolamide") ) AND ( LIMIT-TO(DOCTYPE,"ar") ) AND ( LIMIT-TO(PUBYEAR,2025) OR LIMIT-TO(PUBYEAR,2024) OR LIMIT-TO(PUBYEAR,2023) OR LIMIT-TO(PUBYEAR,2022) OR LIMIT-TO(PUBYEAR,2021) ) AND ( LIMIT-TO(LANGUAGE,"English") )
Web of Science	((TS=("acute heart failure" OR "acute decompensated heart failure" OR "ADHF")) AND TS=("furosemide" OR "loop diuretic")) AND TS=("continuous infusion" OR "continuous intravenous" OR "bolus" OR "intermittent" OR "sequential nephron blockade" OR "SGLT2 inhibitor" OR "dapagliflozin" OR "empagliflozin" OR "thiazide" OR "hydrochlorothiazide" OR "metolazone" OR "acetazolamide")) AND PY=(2021-2025) AND LA=(English) AND DT=(Article)
Cochrane Library	("acute heart failure" OR "acute decompensated heart failure" OR "ADHF") AND (furosemide OR "loop diuretic") AND ("continuous infusion" OR "continuous intravenous" OR bolus OR intermittent OR "sequential nephron blockade" OR "SGLT2 inhibitor" OR dapagliflozin OR empagliflozin OR thiazide OR hydrochlorothiazide OR metolazone OR acetazolamide) (Word variations have been searched) with Publication Year from 2021 to 2025 in Cochrane Reviews, Cochrane Protocols, and Trials
ClinicalTrials.gov	(acute heart failure OR acute decompensated heart failure OR ADHF) AND (furosemide OR loop diuretic) AND (continuous infusion OR bolus OR sequential nephron blockade OR SGLT2 inhibitor OR dapagliflozin OR empagliflozin OR thiazide OR hydrochlorothiazide OR metolazone OR acetazolamide)
